# Ultrasonic engineering of bovine serum albumin nanoparticles for high internal phase Pickering emulsions: Interfacial behavior, microstructural evolution and stabilization enhancement

**DOI:** 10.1016/j.ultsonch.2025.107543

**Published:** 2025-08-28

**Authors:** Liyuan Ma, Jie Li, Yixiang Liu, Jie Zheng

**Affiliations:** a College of Ocean Food and Biological Engineering, Jimei University, Xiamen, Fujian 361021, People's Republic of China; b School of Chemistry and Chemical Engineering, Chongqing University, Chongqing 401331, People's Republic of China

**Keywords:** Pickering emulsions, Ultrasonic treatment, Bovine serum albumin, Interfacial adsorption, Microstructure, Stability

## Abstract

This study investigated the influence of ultrasonic treatment on the physicochemical properties of bovine serum albumin (BSA) and its applicability in stabilizing high internal phase Pickering emulsions (HIPPEs). Under optimized sonication conditions (250 W, 12 min), stable ultrasonically modified BSA (UBSA) particles were generated, exhibiting a small particle size (41.39 nm), a polydispersity index < 0.17, a higher absolute zeta potential (> 20 mV), and favorable wettability (three-phase contact angle: 78.71°), accompanied by reduced surface hydrophobicity. Intrinsic fluorescence spectra and circular dichroism (CD) analysis confirmed that ultrasonication altered the secondary structure of BSA, leading to the exposure of hydrophobic groups. Dynamic interfacial tension and adsorption kinetics analyses revealed that UBSA particles exhibited lower interfacial tension and significantly higher diffusion coefficient (*K_diff_*) and penetration coefficient (*K_p_*) than native BSA, indicating enhanced diffusion and adsorption capabilities of UBSA at the oil–water interface. Rheological analyses demonstrated that UBSA-stabilized HIPPEs possessed higher viscosity and larger storage (*G′*) and loss (*G″*) moduli. Optical and confocal laser scanning microscopy confirmed the successful formation of HIPPEs at UBSA concentrations ≥ 1.0 % (w/v). UBSA-stabilized HIPPEs displayed a reduced droplet size (10.59 µm) and a more densely packed droplet structure, which conferred enhanced resistance against droplet coalescence compared to emulsions stabilized by native BSA. Moreover, stability assessments indicated that the centrifugal, freeze–thaw and storage stability of the prepared HIPPEs were significantly improved. Importantly, UBSA-based HIPPEs serving as a delivery vehicle also effectively enhanced the thermal processing stability of β-carotene. The findings demonstrate the potential of ultrasound-modified BSA nanoparticles as effective stabilizers for HIPPEs, providing valuable insights for the development of healthy and safe food-grade emulsion systems.

## 1 Introduction

High internal phase emulsions (HIPEs), concentrated emulsion systems with an internal phase volume fraction exceeding 74 %, are widely used in the food industry due to their tunable semi-solid behavior, smooth oral texture, and high loading capacity for fat-soluble bioactives [1]. Compared to HIPEs stabilized by conventional surfactants, high internal phase Pickering emulsions (HIPPEs) stabilized by solid particles exhibit superior stability and have become a major research focus. HIPPEs demonstrate enhanced resistance to Ostwald ripening and coalescence relative to surfactant-stabilized equivalents, attributable to the high desorption energy barrier of solid particles [2]. Although solid particles such as silica, titanium dioxide, and clay exhibit excellent potential for stabilizing HIPPEs in food applications, their inherent limitations—including difficult biodegradability and poor biocompatibility—remain significant drawbacks [3]. Consequently, developing natural, eco-friendly, food-grade particles for HIPPEs stabilization has emerged as a critical research priority.

Water-soluble proteins (e.g., soy protein, whey protein), prevalent nutritional biomolecules in food systems, exhibit significant potential for constructing HIPPEs due to their amphiphilicity, environmental benignity, favorable biocompatibility, and modifiability [4]. However, the practical application of partially native protein particles in HIPPEs is often constrained by inherent limitations, including insufficient hydrophobicity, inadequate stability, and suboptimal interfacial properties [5,6]. To overcome these constraints, molecular modification strategies such as cross-linking, compounding, and glycosylation have been extensively employed to enhance the interfacial behavior and functional performance of protein particles. For instance, non-covalent modification of pea protein isolate (PPI) with high methoxyl pectin (HMP) effectively enhanced the oil–water interfacial adsorption properties of PPI across varying pH conditions, thereby enabling the formation of highly stable HIPPEs resistant to pH-induced destabilization [7]. Chitosan-caseinophosphopeptides nanocomplexes endowed HIPPEs with exceptional viscoelasticity, self-supporting capacity, and thixotropy [8]. Furthermore, pea protein isolate-fucoidan conjugates synthesized via the Maillard reaction exhibited excellent emulsifying capacity, with their stabilized emulsions demonstrating resistance to ionic strength and protective effects against encapsulated curcumin oxidation [9]. Although these covalent conjugation or polysaccharide/polyphenol complexation strategies enhanced interfacial activity and functional properties for HIPPEs stabilization, they introduce manufacturing complexity and potential chemical residue risks. Moreover, these approaches may compromise the native nutritional attributes of proteins, contradicting clean-label food principles that demand “minimally processed, all-natural” ingredients. Therefore, simpler, efficient, green, and non-chemically intervening physical modification techniques that directly improve the interfacial properties of single-component food proteins are urgently needed.

Ultrasound, a promising green physical processing technology, can induce protein conformational changes, promote hydrophobic group exposure, and enhance interfacial activity via cavitation effects [10]. Ultrasound has produced spherical rice peptide nanoparticles with small size, narrow distribution, and low interfacial tension [11]. Furthermore, Jiang et al. [2] reported that ultrasonic treatment (520 W, 90 min) reduced silkworm pupa protein particle size to 152.67 nm and increased surface hydrophobicity, enabling the modified proteins to stabilize oil-in-water HIPPEs with intestinal release properties. Similarly, sonication (400 W, 7 min) modified yeast protein particles exhibited a more relaxed molecular conformation and near-neutral wettability (three-phase contact angle of 88.91°), stabilizing HIPPEs characterized by optimal interfacial protein adsorption and a homogeneous three-dimensional protein network [12]. Based on existing literature, current research on protein adsorption at the oil–water interface primarily focuses on poorly water-soluble or alcohol-soluble proteins. However, albumin—a nutritionally complete protein abundant in both plant- and animal-derived foods—has received considerably less attention. This oversight stems from the inherent properties of natural albumin in its native conformation: its high water solubility impedes effective interactions with the oil–water interface, thereby limiting its application as a stabilizer for HIPPEs.

To address this technical challenge in developing albumin-based Pickering emulsifiers, bovine serum albumin (BSA) was selected as a representative model. This study systematically investigates ultrasound-mediated physicochemical modifications of BSA to evaluate its potential as a Pickering emulsion stabilizer. The effects of power and duration on BSA particle wettability, size, size distribution, zeta potential, turbidity, intrinsic fluorescence, and surface hydrophobicity were analyzed to optimize treatment parameters. Ultrasonically modified BSA was subsequently evaluated for HIPPEs construction. Interfacial adsorption behaviors at varying particle concentrations were examined. HIPPEs stabilized by different particle concentrations were characterized for visual appearance, droplet size distribution, microstructure, and stability (including high-speed centrifugation, freeze–thaw cycling, and storage). These findings offer new insights into developing protein-based particles for HIPPEs stabilization.

## 2 Materials and methods

### 2.1 Materials

The BSA (≥98 % purity, CAS# 9048–46-8) was obtained from Sigma-Aldrich (St. Louis, MO, USA). Soybean oil was procured from Fujian Xinde Foods Co., Ltd. (Fujian, China). 8-anilino-1-naphthalenesulfonic acid (ANS) and Nile Red were purchased from Shanghai Yuanye Bio-Technology Co., Ltd. (Shanghai, China) and procured from Shanghai Macklin Biochemical Co., Ltd. (Shanghai, China), respectively. Nile blue A was acquired from Adamas beta Chemical Reagent Co., Ltd. (Shanghai, China). All other chemicals were of analytical grade.

### 2.2 The preparation of ultrasound-modified bovine serum albumin (UBSA)

The ultrasound protocol was adapted from Cheng et al. [12] with modifications. The BSA powder was dissolved in phosphate buffer solution (pH 7.0) to prepare a 2.0 % (w/v) dispersion. The mixture was magnetically stirred until homogeneous and then stored at 4 °C overnight. Prior to sonication, the solution was equilibrated to ambient temperature (25 ± 1°C). Subsequently, 10 mL aliquots of the BSA solution were subjected to ultrasonic treatment in 20 mL flat-bottomed vials (∅ 24.0 mm) using an ultrasonic processor (SCIENZ-ⅡD, Xinzhi Biotechnology Co., Ltd., Ningbo, China) equipped with a 6 mm diameter probe. Sonication was performed in pulse mode (2 s on / 2 s off). Ultrasonic parameters were optimized as follows: (1) Fixed time (10 min): power varied at 0, 100, 150, 200, 250, 300, 350, and 400 W; and (2) fixed power (250 W): time varied at 9, 11, 12, 13, 14, and 15 min. Following ultrasonication, aliquots were collected for immediate analysis. Residual samples were lyophilized (PD-1A-50, BIOCOOL, Beijing, China) for subsequent HIPPEs preparation. The sample treated under optimal ultrasonic conditions was designated ultrasound-modified bovine serum albumin (UBSA), with untreated BSA solution serving as the control.

### 2.3 Measurement of three-phase contact angle

The three-phase contact angle (*θ*) of protein powders was measured using an optical contact angle goniometer (SDC-200S, Shengding Precision Instrument Co., Dongguan, China) according to established methods [13,14]. Lyophilized BSA and UBSA powders were compressed into thin tablets and placed in quartz dishes filled with soybean oil. A deionized water droplet (2 μL) was dispensed onto the tablet surfaces via microsyringe. After equilibration, droplet images were captured and analyzed with the instrument’s proprietary software.

### 2.4 Particle size, polydispersity index (PDI), and zeta (ζ) −potential measurements

Particle size, PDI, and zeta potential of BSA and UBSA particles were determined according to methods described by Huang et al. [15] and Cheng et al. [12] with appropriate modifications. Analyses were conducted at 25 °C using a Zetasizer Nano-ZS90 (Malvern Instruments Ltd., Malvern, UK). Particle size and PDI were assessed by dynamic light scattering (DLS) at a fixed scattering angle of 90°, while zeta potential values were derived from electrophoretic mobility measurements using the Smoluchowski model. Sample solutions were diluted to 0.1 % (w/v) prior to analysis.

### 2.5 Turbidity determination

Sample solution turbidity was determined by measuring light transmittance at 500 nm using an ultraviolet–visible spectrophotometer (Lambda 265, PerkinElmer Inc., USA) following established analytical protocols [16]. A negative correlation exists between transmittance values and turbidity, where lower transmittance corresponds to higher turbidity. All measurements were performed at 25 °C in triplicate.

### 2.6 Intrinsic fluorescence spectra analysis

Intrinsic fluorescence of BSA and UBSA particles was analyzed using a fluorescence spectrophotometer (Cary Eclipse, Agilent Inc., USA) [17]. Solutions were diluted to 0.50 mg/mL with phosphate buffer (10 mM, pH 7.0). Fluorescence emission spectra (300–500 nm) were recorded at a fixed excitation wavelength of 280 nm using a 5 nm slit width.

### 2.7 Determination of surface hydrophobicity

Surface hydrophobicity was determined using 8-anilino-1-naphthalenesulfonic acid (ANS) as a fluorescent probe, adapted from Jiang et al. [2]. Samples were diluted to serial concentrations (0.01, 0.02, 0.03, 0.04, and 0.05 % w/v) with 10 mM phosphate buffer (pH 7.0). Subsequently, 20 μL of 8 mM ANS solution (prepared in identical buffer) was added to 4 mL protein aliquots. Mixtures were incubated in darkness for 15 min at 25 °C. Fluorescence was measured (Cary Eclipse, Agilent Inc., USA) at excitation 390 nm/emission 470 nm. Surface hydrophobicity index (*H_0_*) was calculated as the slope of the linear regression between fluorescence intensity and protein concentration.

### 2.8 Interfacial adsorption analysis

Dynamic interfacial tension at oil–water interfaces was determined using a tensiometer (DCAT15, DataPhysics Instruments GmbH, Germany) equipped with a Wilhelmy plate, following established methodologies with minor modifications [[18], [19], [20]]. Soybean oil (low-density phase) was introduced into a glass beaker and the platinum plate was suspended. The instrument automatically calculated the oil phase buoyancy factor, after which the beaker was replaced. Subsequently, the aqueous phase (high-density liquid) was introduced first, followed by soybean oil addition when instrument-prompted. Measurements were conducted over 3600 s to monitor interfacial tension evolution. Interfacial pressure evolution (*π*) during membrane adsorption was quantified via Equation (1):

(1) π_t_ = γ_buffer t_ – γ_t_

where *π_t_* denotes interfacial pressure (mN·m^−1^) at time *t* (s), with *γ_buffer t_* and *γ_t_* representing interfacial tensions for PBS buffer-soybean oil and protein suspension-soybean oil systems, respectively.

Nanoparticle dynamics at the oil–water interface progressed through three kinetic regimes: diffusion, permeation, and structural reorganization. Initial diffusion behavior was modeled using Equations (2), (3):

(2) π = 2*C_0_K_B_T*(*Dt*/3.14)^0.5^

(3) *K_diff_* = 2*C_0_K_B_T*(*D*/3.14)^0.5^

Here *C_0_* is protein suspension concentration; *K_B_*, *T, D,* and *t* correspond to Boltzmann's constant, absolute temperature, diffusion coefficient, and adsorption duration. *K_diff_* specifically describes colloidal nanoparticle diffusion kinetics.

Permeation (*Kp*) and rearrangement (*Kr*) rate constants were derived from first-order kinetics (Equation (4):

(4) ln[(π*_3600_*-π*_t_*)/(π*_3600_*-π*_0_*)] = -*Kt*

with *π_3600_*, *π_0_*, *and π_t_* indicating interfacial pressures at terminal (3600 s), initial (0 s), and intermediate timepoints. *K* represents the rate constant for the permeation and rearrangement periods.

### 2.9 Circular dichroism (CD) analysis

Secondary structural changes in the protein were analyzed by CD spectroscopy according to previous studies [21]. Briefly, BSA samples were diluted to 0.10 mg/mL using 10 mM phosphate-buffered saline (PBS, pH 7.4). CD spectra were recorded from 190 to 260 nm at 25 °C using a spectropolarimeter (Chirascan, Applied Photophysics Ltd., Leatherhead, UK) equipped with a 0.1 cm pathlength quartz cell, with settings of 120 nm/min scan speed, 1 nm bandwidth, and three accumulations per measurement.

### 2.10 Preparation of HIPPEs

HIPPEs with 75 % (v/v) oil phase were prepared following a published method with slight modifications [12]. BSA or UBSA solutions were mixed with soybean oil (1:3 v/v) using a high-speed digital homogenizer (HR-500D, Shanghai Huxi Industrial Co., Ltd., China) at 15,000 rpm for 2 min. Protein concentrations of 0.10, 0.50, 1.00, 2.00, 3.00, 4.00, and 5.00 % (w/v) were evaluated for stabilization efficacy. Fresh HIPPEs analyses were completed within 2 h of preparation.

### 2.11 Optical microscopy (OPM) observation and droplet size analysis

Optical microscopy (OPM) observations followed Xu et al. [22] with modifications. A minute aliquot of freshly prepared HIPPEs was deposited on a glass slide, gently covered with a coverslip to prevent emulsion aggregation and air bubble entrapment. Emulsion microstructures were examined at 40 × magnification using an inverted fluorescence microscope (Ts2R-FL, Nikon, Japan). Surface-average droplet diameter (D̄) was quantified from micrographs using image analysis software (Nano Measurer 1.2, Fudan University, China). For statistical reliability, three random micrographs per sample were analyzed, with discernible droplets measured for planar diameter.

### 2.12 Confocal laser scanning microscopy (CLSM) observation

HIPPEs microstructures were analyzed per established methodology [23] using a confocal laser scanning microscope (TCSSP8, Leica, Germany) with 40 × objective. Prior to imaging, samples were stained with a fluorescent dye solution containing equal volumes of Nile Blue A (0.10 wt% in distilled water) and Nile Red (0.10 wt% in isopropyl alcohol), followed by dark incubation at ambient temperature. Nile Blue A labeled the aqueous phase while Nile Red stained the oil phase. Stained samples were mounted on slides with glycerol-sealed edges. Imaging employed a helium–neon laser (633 nm, Nile Blue A) and argon-krypton laser (488 nm, Nile Red).

### 2.13 Rheological measurement

Rheological measurements were performed on a MCR302 Modular Compact Rheometer (Anton Parr, Austrian) equipped with 60-mm parallel plates (1.0 mm gap) via oscillatory and steady shear modes, consistent with prior methodology [14]. All tests were conducted at 25 °C, and the sample was allowed to equilibrate on the instrument for 3 min prior to testing. The following procedures were carried out:

(1) Dynamic viscoelasticity test: The apparent viscosity of the emulsion sample was measured as a function of shear rate within the range of 0.1 to 100 s^−1^. The viscosity curve was fitted using a power-law model.

(2) Frequency sweep test: Initially, an amplitude scan was performed at an angular frequency of 10 rad/s and strain ranging from 0.01 % to 100 % to determine the linear viscoelastic region. Subsequently, the dynamic viscoelasticity of the emulsion samples was measured within the linear viscoelastic region at frequencies of 0.1–100 rad/s and a strain of 1.0 %, determining the storage modulus (G′) and loss modulus (*G“*).

### 2.14 Centrifugal stability measurement

Centrifugal stability of HIPPEs was assessed following Wang et al. [24] with minor modifications. Aliquots (2.5 g) of freshly prepared HIPPEs in 5 mL centrifuge tubes were centrifuged at 10,000 rpm for 20 min, then photographed for stability evaluation.

### 2.15 Freeze-thaw stability determination

Freeze-thaw stability was evaluated according to the method of Chen et al. [25]. HIPPEs were stored isothermally at −20 °C for 24 h, then thawed at 25 °C for 4 h. This cycle was repeated identically until three complete freeze–thaw cycles were achieved.

### 2.16 Storage stability measurement

Storage stability was determined following a published method [5] with minor modifications. HIPPEs were stored at ambient temperature for 30 days, with stability assessed through visual appearance and microstructure analysis. Microstructural characterization used an optical microscope (Ti-S, Nikon, Tokyo, Japan). Droplet mean diameters were calculated from micrographs using image analysis software (Nano Measurer 1.2, Fudan University, China).

### 2.17 Thermal processing stability analysis

To evaluate the protective effect of HIPPEs against the thermal degradation of β-carotene during food processing, samples were subjected to simulated pasteurization (65 °C) and thermal cooking (100 °C) conditions. Specifically, samples were heated in a water bath at 65 °C for 30 min and 100 °C for 10 min, respectively. For comparison, unencapsulated β-carotene dispersed directly in oil served as the control. Following thermal treatment, the retention rate of β-carotene was determined [26]. Briefly, 0.5 g of the emulsion was mixed with 1 mL of ethanol and 4 mL of *n*-hexane. The mixture was vigorously vortexed for 2 min to facilitate extraction. The *n*-hexane phase containing the extracted β-carotene was then collected. This extraction procedure was repeated three times using fresh solvent each time. All *n*-hexane fractions were combined and diluted to a fixed volume in a volumetric flask. The absorbance of the combined extract was measured at 450 nm using a UV–Vis spectrophotometer. The β-carotene concentration was quantified based on a pre-established standard calibration curve (concentration vs. absorbance). The β-carotene retention rate (%) was calculated as follows:

(5) Retention rate (%) = (*C* / *C_0_*) × 100

where: *C_0_* = Initial β-carotene content in the sample (prior to heating); *C* = β-carotene content in the sample after thermal treatment.

### 2.18 Statistical analysis

All measurements were performed in triplicate (three independent replicates per sample), with results expressed as mean ± standard deviation (SD). The SPSS Statistics 23.0 (SPSS Inc., Chicago, IL, USA) was used to conduct a one-way analysis of variance (ANOVA) on the data with Duncan’s multiple range test (*P* < 0.05). Data visualizations were generated using Origin 2025 (OriginLab Corporation, Northampton, MA, USA).

## 3 Results and discussion

### 3.1 Effect of ultrasonic power on physicochemical characteristics of BSA

**Fig. 1:**
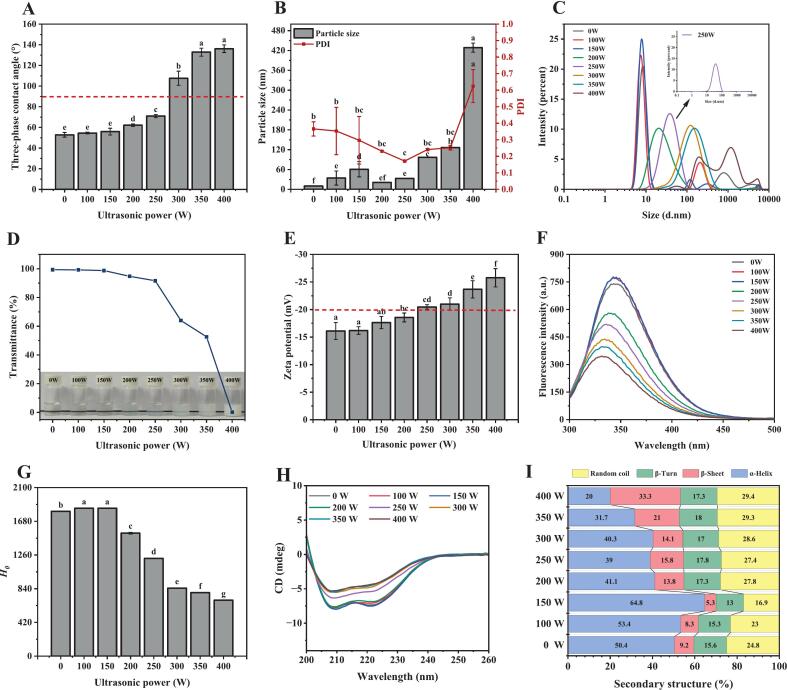
Physicochemical properties of BSA after ultrasonic power treatments: (A) Three-phase contact angle, (B) particle size and PDI, (C) particle size distribution, (D) turbidity, (E) zeta potential, (F) intrinsic fluorescence spectroscopy, (G) surface hydrophobicity, (H) circular dichroism (CD), and (I) protein secondary structure changes. Different letters indicate statistically significant differences (*P* < 0.05).

Particle physicochemical properties—including wettability, size, and surface charge—significantly influence adsorption and accumulation behavior at oil–water interfaces [12]. The three-phase contact angle (*θ*), a critical wettability parameter, dictates interfacial orientation and emulsion classification [27]. As shown in Fig. 1**A**, native BSA exhibited hydrophilic character (*θ* = 52.87 ± 2.28°). Ultrasonication progressively modified wettability: a low-intensity treatment (100–150 W) maintained statistically unchanged *θ* values (*P* < 0.05), while higher power (200–250 W) increased *θ* to 62.13–70.93°, enhancing oil-wetting capacity. This aligns with ultrasonicated rice peptide nanoparticles (*θ* = 72.1°) that effectively stabilized oil-in-water emulsions [11]. Notably, excessive power (> 300 W) induced lipophilic dominance (*θ* > 107.59°), exceeding the near-neutral wettability threshold (*θ* ≈ 90°) essential for interfacial adsorption and steric barrier formation [28]. These findings establish controlled ultrasonication as an effective strategy for optimizing BSA wettability.

Protein particles with small size and low polydispersity (PDI ≤ 0.5) preferentially adsorb at oil/water interfaces, facilitating Pickering emulsion formation [26]. Fig. 1**B-C** reveal ultrasonication exerted biphasic control over particle size evolution. At 0–150 W, particle size increased progressively with disordered multi-peak distributions. Beyond this range, higher power first reduced then increased particle size and PDI, accompanied by transition to unimodal distributions. Crucially, at 250 W, BSA displayed a monodisperse distribution (33.24 ± 0.08 nm), low PDI (0.17 ± 0.01), minimal turbidity (transmittance > 91 %), and high electrostatic stability (|ζ-potential| > 20 mV; Fig. 1**B-E**), confirming stable nanoparticle formation [29].

Intrinsic fluorescence spectroscopy probed tertiary structural rearrangements. Surface hydrophobicity (*H_0_*), reflecting exposure of hydrophobic groups, critically determines protein adsorption at interfaces [30]. Mild ultrasonication (≤150 W) increased fluorescence intensity and *H_0_* (Fig. 1**F-G**), likely due to cavitation-induced molecular unfolding and exposure of buried tryptophan residues [31]. Conversely, higher power (200–400 W) caused a gradual blue-shift in the maximum fluorescence peak position of BSA, indicating reduced polarity around tryptophan residues, alongside decreased *H_0_*. This likely results from ultrasonication-induced protein collisions and aggregation, burying previously exposed hydrophobic groups [32]. Analogous fluorescence and *H_0_* reductions were reported for silkworm pupa protein under ultrasonication [2]. As shown in Fig. 1**H-I**, CD analysis further demonstrated that ultrasonication induced conformational changes in BSA. The alterations in α-helix and β-sheet content became increasingly pronounced with escalating ultrasonic power. At 250 W treatment, the α-helix content decreased from 50.4 % (untreated BSA) to 39.0 %, while β-sheet content increased from 9.2 % to 15.8 %. When power was elevated to 400 W, more significant secondary structural reorganization occurred: α-helix content further decreased to 20.0 %, and β-sheet content rose substantially to 33.3 %. Collectively, optimized ultrasonication (250 W) generates monodisperse, stable BSA particles with balanced wettability and enhanced interfacial activity.

### 3.2 Effect of ultrasonic time on physicochemical properties of BSA

**Fig. 2:**
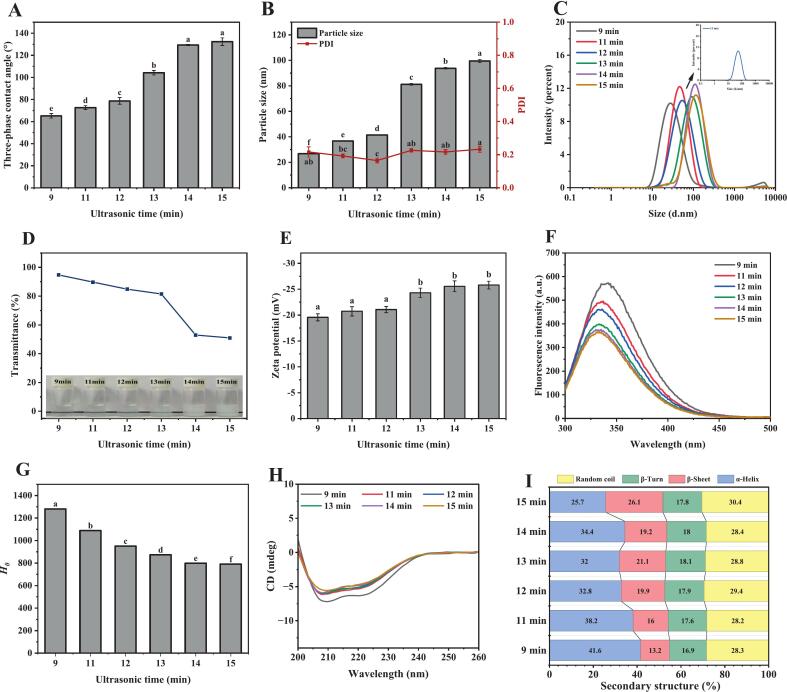
(A) Three-phase contact angle, (B) particle size and PDI, (C) particle size distribution, (D) turbidity, (E) zeta potential, (F) intrinsic fluorescence, (G) surface hydrophobicity, (H) circular dichroism (CD), and (I) protein secondary structure changes of BSA after different ultrasonic time treatments. Note: Different letters on the bars indicate significant differences (*P* < 0.05).

Near-neutral wettability promotes efficient particle accumulation at the oil–water interface, establishing robust steric hindrance that inhibits droplet coalescence [33]. As shown in Fig. 2**A,** the contact angle (*θ*) of bovine serum albumin (BSA) particles increased with extended ultrasound duration, indicating enhanced wettability toward the oil phase. Notably, after 12 min of sonication, the *θ* value of BSA particles reached 78.71 ± 3.02°, approaching 90°. This near-neutral wettability signifies strong interfacial adsorption capacity and emulsion-stabilizing potential, facilitating the stabilization of oil-in-water Pickering emulsions. A similar trend was observed for spirulina proteins, whose *θ* increased from 65.5° to 84.3° under ultrasonic treatment [34]. Furthermore, prolonged sonication time initially led to a gradual increase in particle size (≤12 min), followed by a sharp increase (> 12 min), while maintaining a monomodal size distribution with a PDI < 0.25 (Fig. 2**B-C**). We attribute this size evolution to the gradual intensification of the ultrasonic field, which increases the frequency of interparticle collisions and promotes aggregation [12]. This observation is further corroborated by the turbidity results (Fig. 2**D**).

Additionally, the absolute zeta-potential value of BSA increased with sonication time (Fig. 2**E**), implying enhanced electrostatic repulsion between particles. This increased repulsive force contributes to the resistance against particle aggregation [35].

Changes in the intrinsic fluorescence of tryptophan residues reflect alterations in their side-chain microenvironments and protein conformational rearrangement [36], whereas surface hydrophobicity (*H_0_*) influences particle adsorption capacity at the oil–water interface [37]. As shown in Fig. 2**F-G**, ultrasonic treatment between 9 and 15 min induced a decrease in both fluorescence intensity and *H_0_* for BSA. This decrease can be explained by the re-aggregation of exposed hydrophobic groups through intramolecular hydrophobic interactions under prolonged cavitation, leading to their reburial within the protein interior [32]. This finding aligns with a report by Cheng et al. [12], where continuous sonication (11 min) reduced the *H_0_* of yeast proteins. Analysis of protein secondary structure changes via CD spectroscopy (Fig. 2H-I) revealed progressively more pronounced conformational alterations in BSA with prolonged ultrasonication time at fixed 250 W power. Consistent with power-dependent effects, ultrasound-induced structural reorganization primarily involved α-helix and β-sheet elements. Following 9-min ultrasonication at 250 W, α-helix and β-sheet contents measured 41.6 % and 13.2 %, respectively. When exposure time increased to 12 min at identical power, these values shifted to 32.8 % (α-helix) and 19.9 % (β-sheet). This trend intensified significantly after 15 min of treatment, ultimately yielding 25.7 % α-helix and 26.1 % β-sheet content. Consequently, after 12 min of 250 W ultrasonic treatment, the resulting protein particle system exhibited structural stability, small particle size, and near-neutral wettability, exhibiting optimal characteristics as a Pickering emulsion stabilizer.

### 3.3 Formation and characterization of HIPPEs

**Fig. 3:**
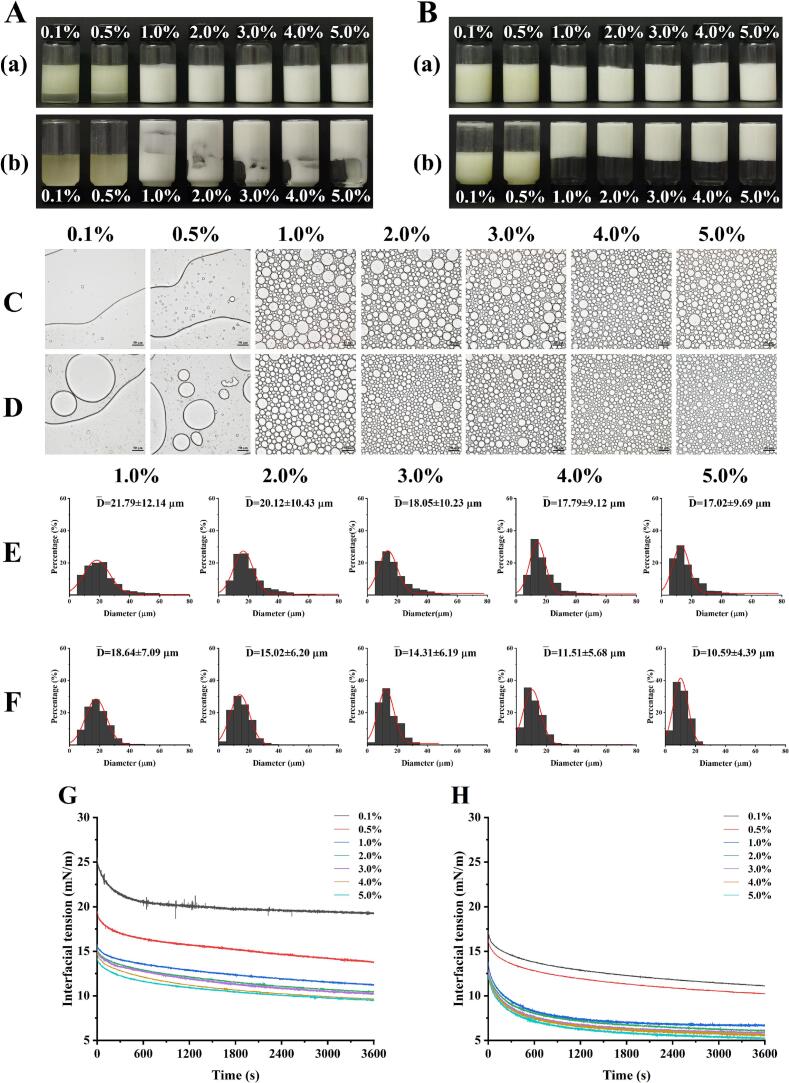
Characterization of stabilized high internal phase Pickering emulsions (HIPPEs) at different protein particle concentrations. Visual appearance of HIPPEs stabilized by (A) BSA and (B) UBSA particles; optical microscopy images of HIPPEs stabilized by (C) BSA and (D) UBSA particles; droplet size distribution of HIPPEs stabilized by (E) BSA and (F) UBSA particles; and dynamic interfacial tension at the oil–water interface for (G) BSA and (H) UBSA versus time. Notes: (a) Upright position and (b) inverted position in panels (A) and (B); scale bar in panels (C) and (D) represents 50 μm.

The minimum particle concentration required to stabilize HIPPEs serves as a critical indicator of colloidal particles’ efficacy as stabilizers [38]. As shown in Fig. 3**A-B**, neither native BSA nor UBSA successfully formed stable emulsions at lower concentrations (0.10 %–0.50 %, w/v), with immediate and distinct phase separation observed due to insufficient interfacial coverage. However, upon increasing the particle concentration to 1.00 % (w/v), a significant difference emerged: while native BSA formed only a semi-fluid emulsion, UBSA-stabilized HIPPEs exhibited rigid, self-supporting behavior without flow upon inversion. This marked change indicates the formation of a three-dimensional gel-like network structure within the continuous phase, facilitated by the enhanced interfacial activity of UBSA particles.

**Fig. 4:**
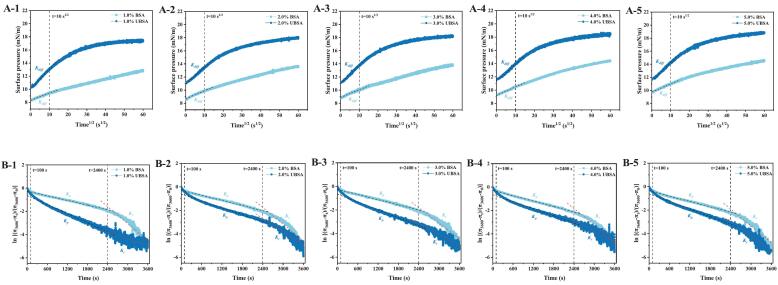
Fitting of the surface pressure (π) during the first diffusion process at the oil–water interface according to Equation (2) (A-1 to A-5) and fitting of π during the late adsorption stage at the oil–water interface according to Equation (4) (B-1 to B-5).

Microstructural characterization is essential for elucidating the stability mechanisms of emulsion systems. Consistent with the macroscopic observations, optical microscopy revealed virtually no stable droplets in systems prepared with either BSA or UBSA at 0.10 %–0.50 % (w/v) concentrations (Fig. 3**C-D**), confirming that their inability to effectively adsorb at the oil–water interface. In contrast, HIPPEs formulated at particle concentrations of 1.00 %–5.00 % (w/v) displayed densely packed, regular spherical droplets. Notably, the mean droplet size decreased progressively with increasing particle concentration (Fig. 3**C-F**), consistent with established Pickering stabilization principles where higher particle loadings enable finer droplet subdivision. Furthermore, HIPPEs stabilized by UBSA consistently exhibited a narrower droplet size distribution and smaller, more uniform droplet dimensions compared to those stabilized by native BSA at equivalent concentrations. This superior microstructure homogeneity directly correlates with enhanced emulsion stability against coalescence. Collectively, these results demonstrate that ultrasonic modification significantly enhances the functionality of BSA particles, rendering UBSA a markedly more effective Pickering stabilizer for HIPPEs.

To kinetically analyze the role of protein particles in emulsion formation and stability, we investigated the relationship between protein concentration and interfacial adsorption behavior (Fig. 3**G-H**). Protein adsorption at the oil–water interface occurred in two distinct stages. During the initial stage (< 2 min), particles rapidly adsorbed to the interface, causing a sharp decline in interfacial tension. Notably, UBSA reduced interfacial tension more rapidly than native BSA, which we attribute to UBSA’s near-neutral wettability enhancing diffusion kinetics [12]. Subsequently, interfacial tension reached a dynamic plateau as adsorption approached saturation. This transition likely results from interfacial crowding effects where adsorbed BSA/UBSA particles generate steric hindrance and electrostatic repulsion, thereby limiting further adsorption [39,40]. Furthermore, the equilibrium interfacial tension exhibited strong concentration dependence for both protein types, confirming particle concentration as a critical modulator of interfacial activity. At 0.1–0.5 % concentrations, relatively high interfacial tensions indicated poor HIPPE stabilization by either BSA or UBSA. When concentrations of BSA/UBSA reached ≥ 1.0 %, both systems showed marked reductions in equilibrium interfacial tension. Consequently, we will conduct a comparative analysis of BSA/UBSA adsorption kinetics at oil–water interfaces across 1.0–5.0 % concentrations and their corresponding HIPPE stabilization effects in subsequent experiments.

### 3.4 Adsorption kinetics

Particle adsorption at interfaces progresses through three sequential kinetic stages: diffusion, permeation, and structural rearrangement [20]. During the initial adsorption phase (characterized by low surface pressure), we employed the Ward-Tordai equation to quantify adsorption kinetics [18]. The linear correlation between surface pressure and *t^1/2^* (0–10 s) indicates diffusion-controlled adsorption for both BSA and UBSA, with slopes corresponding to their diffusion rate constants (*K_diff_*) **(**Fig. 4A-1 **to A-5)**. Significantly, across the 1.0–5.0 % concentration range, UBSA consistently exhibited higher *K_diff_* values than native BSA at equivalent concentrations (*P* < 0.05; Table 1). For instance, at 3.0 % concentration, *K_diff_* values reached 0.125 mN⋅m^−1^⋅s^−0.5^ for BSA versus 0.256 mN⋅m^−1^⋅s^−0.5^ for UBSA, respectively. These results confirm UBSA's enhanced initial adsorption kinetics at oil–water interfaces relative to native BSA.

**Table 1:** Diffusion coefficients (*K_diff_*), penetration coefficients (*K_p_*) and reorganization coefficients (*K_r_*) for BSA/UBSA at different concentrations, respectively.

Samples	*K_diff_* (mNm^−1^ s^−0.5^)	*K_p_* × 10^-4^ (s^−1^)	*Kr* × 10^-4^ (s^−1^)
1.0 % BSA	0.111 ± 0.001^a^	6.684 ± 0.004^a^	21.800 ± 0.083^d^
2.0 % BSA	0.121 ± 0.001^b^	7.250 ± 0.005^b^	22.900 ± 0.090^d^
3.0 % BSA	0.125 ± 0.001^b^	7.312 ± 0.004^b^	23.700 ± 0.109^e^
4.0 % BSA	0.126 ± 0.001^b^	7.761 ± 0.003^c^	23.900 ± 0.102^e^
5.0 % BSA	0.132 ± 0.001^c^	7.570 ± 0.005^c^	24.400 ± 0.107^f^
1.0 % UBSA	0.249 ± 0.002^d^	9.633 ± 0.010^d^	9.600 ± 0.086^a^
2.0 % UBSA	0.254 ± 0.002^d^	10.000 ± 0.013^e^	15.700 ± 0.063^b^
3.0 % UBSA	0.256 ± 0.001^d^	10.700 ± 0.011^f^	15.700 ± 0.063^b^
4.0 % UBSA	0.258 ± 0.002^d^	11.100 ± 0.012^f^	19.200 ± 0.066^c^
5.0 % UBSA	0.277 ± 0.001^e^	13.000 ± 0.013^g^	19.700 ± 0.082^c^

The data was expressed as the average value ± standard deviation. Different letters in the same column indicate a significant difference (*P* < 0.05). BSA: bovine serum albumin; UBSA: ultrasonically modified BSA.

As adsorption time increases (*t^1/2^ >* 10 s), the surface pressure versus *t^1/2^* relationship deviates from linearity, indicating transition from diffusion-dominated to permeation/rearrangement stages. The ln[(π_3600_ − π_t_)/(π_3600_- π_0_)] versus time plot reveal two linear regions **(**Fig. 4B-1 **to B-5)**, where the first slope corresponds to the permeation rate constant (*K_p_*) and the second to the rearrangement rate constant (*K_r_*), reflecting particle conformational flexibility [41]. For both BSA and UBSA, increasing protein concentration significantly elevates *K_r_* and *K_p_* **(**Table 1**)**. Compared to native BSA, UBSA's enhanced *K_p_* values may be attributed to sonication-induced protein unfolding, which reduces energy barriers for interfacial permeation. Moreover, *K_r_* consistently exceeds *K_p_*, indicating that particle rearrangement contributes more substantially than permeation to interfacial membrane formation [41].

### 3.5 CLSM observation

**Fig. 5:**
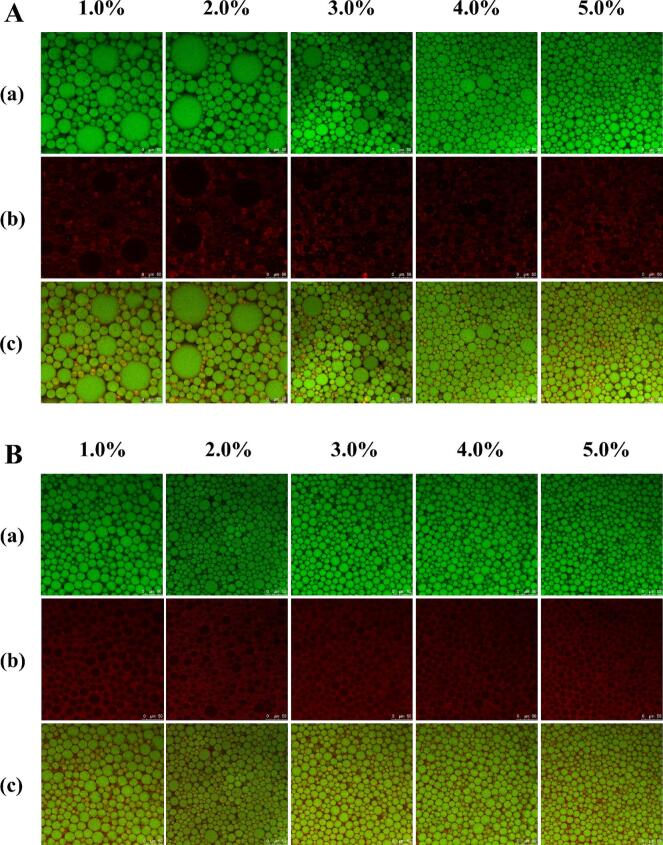
Confocal laser scanning microscopy (CLSM) images of high internal phase Pickering emulsions (HIPPEs) stabilized with (A) BSA and (B) UBSA particles at concentrations of 1.0 %–5.0 % (w/v). Notes: Images were acquired at 40 × magnification; scale bar represents 50 μm. Green fluorescence corresponds to the oil phase, while red fluorescence indicates protein particles adsorbed at the interface. (For interpretation of the references to colour in this figure legend, the reader is referred to the web version of this article.)

The CLSM was employed to elucidate the microstructure of HIPPEs stabilized by native BSA and UBSA particles. As shown in Fig. 5**A-B**, discrete oil droplets (green fluorescence) were enveloped by BSA/UBSA colloidal particles (red fluorescence) within the aqueous continuous phase, confirming the formation of oil-in-water HIPPEs stabilized via Pickering mechanisms. Due to its pronounced hydrophilic character, native BSA exhibited poor interfacial retention, readily desorbing from droplet surfaces into the aqueous phase. This weak adsorption resulted in limited inter-droplet connectivity and the formation of large, polydisperse oil droplets (Fig. 5**A**). In contrast, UBSA nanoparticles demonstrated significantly enhanced interfacial affinity, forming a continuous adsorbed layer on droplet surfaces (Fig. 5**B**). Crucially, the interfacial film density increased progressively with particle concentration, creating a robust steric barrier that effectively inhibited droplet coalescence through combined steric hindrance and interfacial jamming effects [28]. Furthermore, UBSA-stabilized HIPPEs exhibited markedly smaller droplet diameters and superior size uniformity compared to native BSA-stabilized systems, consistent with our earlier droplet size distribution analyses (Section 3.3). Collectively, these microstructural observations demonstrate that ultrasonic modification transforms BSA into an effective Pickering stabilizer. This nanostructured barrier physically impedes droplet coalescence while optimizing droplet size distribution – key determinants of HIPPEs stability.

**Fig. 6:**
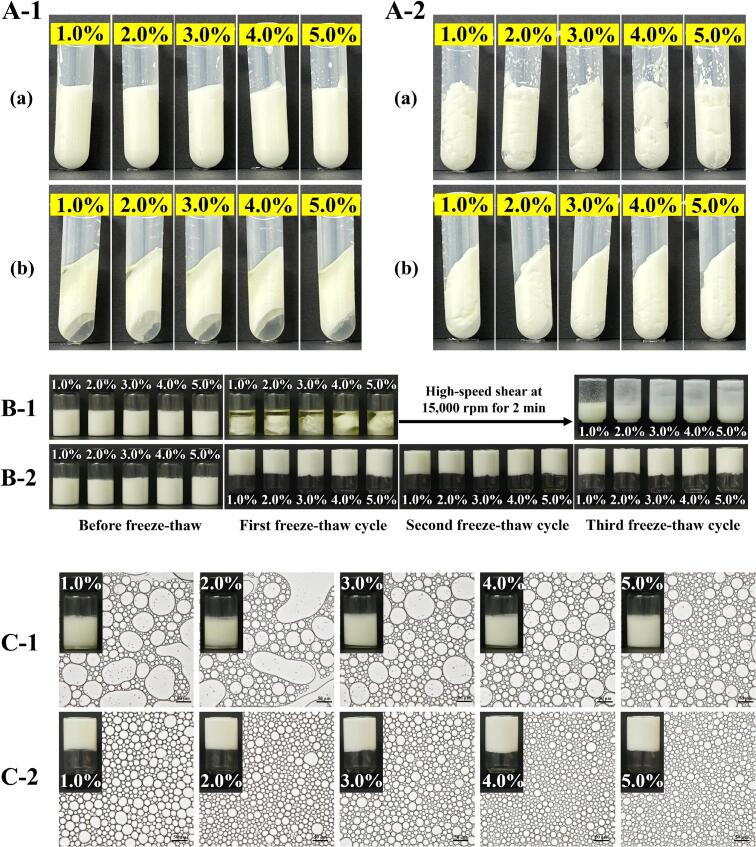
Stability of HIPPEs stabilized with different concentrations (1.0 %–5.0 %) of protein particles. (A) Appearance after centrifugation (10,000 rpm, 20 min): (A-1) BSA-stabilized HIPPEs (a: before; b: after), (A-2) UBSA-stabilized HIPPEs. (B) Appearance before and after freeze–thaw cycles: (B-1) BSA-stabilized HIPPEs, (B-2) UBSA-stabilized HIPPEs. (C) Appearance and optical microscopy (OPM) images after 30 d storage: (C-1) BSA-stabilized HIPPEs, (C-2) UBSA-stabilized HIPPEs. Scale bar: 50 μm (micrographs).

### 3.6 Centrifugal, Freeze-Thaw, and storage stability

The centrifugal, freeze–thaw, and storage stability of HIPPEs stabilized by BSA or UBSA particles at varying concentrations were investigated. As shown in Fig. 6A-1, BSA-stabilized HIPPEs underwent structural disruption with visible oil–water separation after centrifugation, indicating poor centrifugal stability. This instability likely stems from their large, polydisperse oil droplets and insufficient interfacial film strength to resist droplet coalescence during centrifugation [24]. In contrast, UBSA formed a robust interfacial barrier via tight particle stacking, preventing phase separation and enhancing centrifugal stability (Fig. 6A-2**)**. Freeze-thaw stability is critical for evaluating emulsion performance. Ice crystal formation during freezing can penetrate oil droplets, rupturing the interfacial layer and promoting coalescence [42]. BSA-stabilized HIPPEs exhibited severe breakage and oil separation after one freeze–thaw cycle (Fig. 6B-1), while UBSA-stabilized HIPPEs retained structural integrity through three cycles (Fig. 6B-2). Notably, destabilized BSA-based HIPPEs failed to re-emulsify after homogenization, even at low particle concentrations (1.0 %, w/v), confirming irreversible damage.

Storage stability dictates the practicality of emulsion-based products [43]. After 30 days at room temperature, HIPPEs with 1.0 %–4.0 % (w/v) natural BSA showed slight delamination (Fig. 6C-1). Microscopically, these emulsions developed significant droplet aggregation and size growth. Conversely, UBSA-stabilized HIPPEs maintained their inverted structure without macroscopic or microscopic changes (Fig. 6C-2), aligning with reports of enhanced stability in protein–polyphenol-stabilized HIPPEs [44].

### 3.7 Rheological analysis

**Fig. 7:**
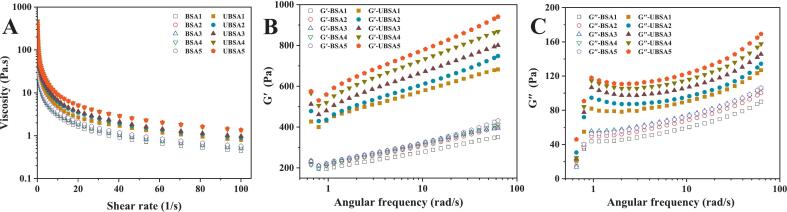
Rheological properties of emulsions: viscosity (A), storage modulus (B), loss modulus (C).

The flow behavior and viscoelasticity of emulsions continuously influence their stability and target applications. The rheological test results for BSA/UBSA-stabilized emulsions are depicted in Fig. 7A-C. All samples exhibited a typical shear-thinning property, characterized by a monotonic decrease in apparent viscosity with increasing shear rate. Apparent viscosity increased with increasing concentrations of BSA and UBSA. Interestingly, after ultrasonication, an increase in apparent viscosity was observed for UBSA-stabilized emulsions (Fig. 7A). High-viscosity dispersed systems can form dense interfacial films, which contribute to emulsion stability [18]. The viscoelastic properties of HIPPEs were further investigated using frequency sweep tests. The storage modulus (*G′*) and loss modulus (*G′′*) of the emulsion samples varied with the angular frequency, as shown in Fig. 7B and C. In both BSA and UBSA systems, *G′* and *G′′* increased monotonically with increasing frequency. Furthermore, BSA/UBSA-stabilized emulsions exhibited *G′* > *G′′*, indicating dominant elastic behavior [20]. Notably, compared with native BSA, both *G′* and *G′′* for UBSA were significantly higher across the measured angular frequency range (0.1–100 rad/s). This increase may be attributed to enhanced interactions between UBSA particles adsorbed on oil droplet surfaces. For instance, at a protein concentration of 3.00 %, G′ and G′′ for BSA increased only from 253.67 Pa and 16.70 Pa to 394.26 Pa and 105.98 Pa, respectively. In contrast, G′ and G′′ for UBSA rose more sharply from 555.85 Pa and 17.66 Pa to 800.79 Pa and 145.61 Pa, respectively, suggesting the formation of a stronger network structure.

### 3.8 Enhanced thermal processing stability of β-Carotene in UBSA-Stabilized HIPPEs

**Fig. 8:**
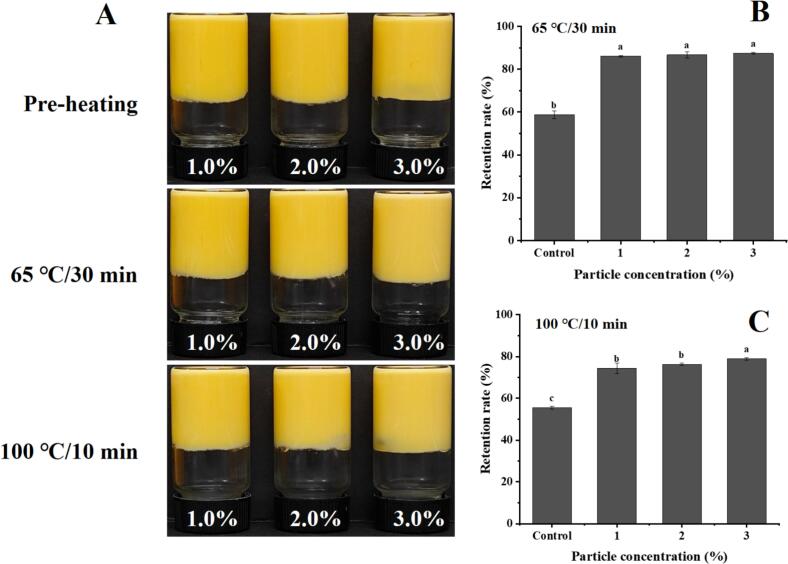
Improvement of the thermal processing stability of β-carotene using UBSA-stabilized HIPPEs. (A) Appearance of β-carotene-loaded HIPPEs before and after thermal treatment. (B) β-Carotene retention under pasteurization conditions (65 °C for 30 min). (C) β-Carotene retention under cooking conditions (100 °C for 10 min). Note: Different letters on the bars indicate significant differences (*P* < 0.05).

Given their efficacy as delivery systems for lipophilic nutrients, this study investigated the ability of UBSA-stabilized high internal phase Pickering emulsions (HIPPEs) to enhance the thermal stability of encapsulated β-carotene. As shown in Fig. 8**A**, the HIPPEs exhibited no significant visual changes following thermal treatment, retaining their characteristic bright orange coloration without detectable phase separation. This indicates the absence of oil droplet aggregation upon heating, demonstrating the inherent thermal stability of the emulsion system. Fig. 8**B-C** compare the retention of β-carotene under simulated pasteurization (65 °C, 30 min) and cooking (100 °C, 10 min) conditions, respectively, within soybean oil (Control) and UBSA-stabilized HIPPEs. β-Carotene dissolved directly in soybean oil showed limited stability, with retention rates of only 58.8 % (pasteurization) and 54.8 % (cooking). Encapsulation within HIPPEs stabilized by UBSA (1.0–3.0 %, w/v) significantly improved (*P* < 0.05) β-carotene retention, achieving values exceeding 85 % and 74 % for pasteurization and cooking processes, respectively. These results clearly demonstrate that UBSA-stabilized HIPPEs effectively protect β-carotene against degradation during thermal processing.

## 4 Conclusions

This study demonstrates that ultrasound-modified bovine serum albumin (UBSA) serves as a highly effective Pickering stabilizer for high internal phase Pickering emulsions (HIPPEs). Sonication at 250 W for 12 min generated UBSA particles with reduced size, narrow polydispersity, high negative surface charge, optimal wettability, and changed molecular conformation. Dynamic interfacial tension and adsorption kinetics analysis revealed UBSA’s superior ability to reduce oil–water interfacial tension, with enhanced diffusion and rearrangement rates. Rheological and structural analyses confirmed that UBSA-stabilized HIPPEs exhibited improved rheological properties, robust inverted self-support, densely packed droplets, and uniform microstructure. Besides, UBSA-based HIPPEs displayed markedly improved centrifugal, freeze–thaw, and long-term storage stability over native BSA-stabilized systems. Critically, UBSA-based HIPPEs as delivery vehicle also effectively enhanced thermal processing stability of β-carotene. These findings establish ultrasound-modified BSA as a natural, sustainable, and efficient stabilizer for HIPPEs, supporting the development of ultrasound-assisted food-grade Pickering emulsions.

## CRediT authorship contribution statement

**Liyuan Ma:** Writing – original draft, Software, Methodology, Investigation, Formal analysis, Data curation. **Jie Li:** Writing – original draft, Software, Methodology, Investigation, Formal analysis, Data curation. **Yixiang Liu:** Writing – review & editing, Supervision, Resources, Project administration, Funding acquisition, Conceptualization. **Jie Zheng:** Writing – review & editing, Supervision, Project administration.

## Declaration of competing interest

The authors declare that they have no known competing financial interests or personal relationships that could have appeared to influence the work reported in this paper.
